# Gun-Shot Wound of the Bladder—Laparotomy and Recovery

**Published:** 1890-06

**Authors:** A. C. Walker

**Affiliations:** Rockdale


DANIEL'S

### TEXAS MEDICAL JOURNAL.

A Representative Organ of the Medical Profession, and an Exponent of Rational Medicine; devoted to the Organization, Advancement and Elevation of the Profession in Texas.

PUBLISHED MONTHLY.—SUBSCRIPTION $2.00 A YEAR.

Vol. V. AUSTIN, JUNE, 1890. No. 12.

### Original Articles.

CONTRIBUTED EXCLUSIVELY TO THIS JOURNAL.

*The Articles in this Department are accepted and published with the understanding that we are not responsible for, nor do we indorse the views and opinions of the writers by so doing.*

For Daniel's Texas Medical Journal.

### GUN SHOT WOUND OF THE BLADDER.—LAPAROTOMY AND RECOVERY.

BY A. C. WALKER, M. D., ROCKDALE.

SURGICAL LITERATURE gives us so little in the way of encouragement in the treatment of gun shot wounds of the bladder that I beg leave to make to the profession a clinical report of a case which lately occurred in my practice.

In Coulson's excellent work on Diseases of the Bladder, he quotes a number of cases of gun shot wounds of the bladder, collected by Dr. Bartels, but in no instance does he advise, as a method of treatment, more than incisions for urinary extravisations, perineal incision and drainage, or a cystotomy in some form.

A gun shot wound invading the peritoneal cavity, penetrating the integrity of the bladder wall, coursing downward to the region of the buttocks; then also an evident existence of an intra-peritoneal urinary escape, as was the indication of wound lesion of the patient whom this report concerns. Little relief for the sufferer could be expected from either a cystotomy or drainage; therefore with the recent brilliant successes in abdominal surgery as a monitor, I at once decided upon such surgical measures as would be recognized in the treatment of intestinal gun shot wounds.

Case.—Frank Taylor, age 21 years, occupation, laborer. In vigorous health; married. On the night of March 2, 1890, while intoxicated and engaged in personal difficulty, he received a gun-shot wound from a thirty-eight calibre pistol, the ball penetrating the abdomen.

A few moments after the injury was inflicted the patient was brought to my office which was only a few blocks away. Upon a casual examination I ascertained that the patient was in extreme shock, the surface bathed in cold perspiration, the radial pulse indistinct, nausea and vomiting, with disposition to pass urine frequently.

Syncope becoming very apparent, I had the patient laid upon the floor of my office, ammonia administered, and caused him to remain in the recumbent position until the temporary disturbance of faintness could pass away. While still upon his back, consciousness again returning, I took occasion to make physical examination of locality and nature of wound. At a point midway between the ant. sup. spinous process of illium and median line, I found an orifice of entrance of a ball seemingly of 38 calibre.

From the direction of the course of the ball, painful retraction of testicles and the desire to constant urination, I at once surmised a wound of the bladder.

I had the patient immediately removed to his home, with the instruction that I would soon follow and perfect a more thorough examination of the injury. Upon seeing the patient the second time I at once catheterized, drawing off four or six ounces of bloody urine.

My diagnosis being confirmed, a catheter was secured in the bladder, the abdominal wound dressed aseptically, an opiate given and the patient dismissed until morning.

The subject being much intoxicated. not competent to decide for himself, I conferred with his family, explaining the gravity of the wound and advising an immediate operation of opening the belly and, if possible, closing the aperture in the bladder, also closing any opening in the bowel, should one exist.

The following morning I was informed by a member of the family that the patient was submissive to any operation however hazardous offering hope for his life. Preparations were at once made for abdominal section. Ten hours after receipt of the injury, with the able assistance of my friends, Drs. Kennard and Horton, the operation was commenced, under the most rigid antiseptic precautions. Ether was the anæsthetic used, which was taken kindly indeed. After carefully shaving the entire abdominal surface, and a free scrubbing with soap and water, followed by a surface bathing of sublimate solution 1 to 1000, the medial abdominal incision was made, extending from a point two inches below the umbilicus to within an inch above the symphysis pubis. Upon reaching the abdominal cavity it was apparent that the cavity was filled with bloody serum, and from its odor I recognized the bladder communication.

The peritoneal cavity was readily cleansed by sponging and drainage, and search begun for the injured viscus.

From the welling up in the pelvic region of a yellowish fluid, I was enabled to locate the lesion of the organ, and quickly secured the edge of the opening with a pair of clamp forceps. Upon examination I found it to correspond to the apex or superior fundus of the bladder. I will say here that at the time the injury occurred his bladder was very much distended with unine, having consumed a quantity of fluids and not having evacuated the bladder for several hours. The aperture in the bladder was held open by the aid of tenacula, and a careful paring of the ragged edges made fresh. Without removing the hold of the tenacula, the process of closing the wound was commenced and completed by the use of a very fine curved needle armed with thoroughly aseptic silk, after the method of Lembert's intestinal suture, which includes the serous and muscular coats, omitting the mucous membrane, bringing out the point of the needle a few lines from the wound margin, and entering the point on side about same distance from the margin.

Sutures thus introduced and made fast invert the wound border, bringing the serous surfaces in close union, sealing securely the perforation.

Six very fine aseptic silk sutures were used and placed not further apart than one-eighth of an inch. I then made search for intestinal wounds; none being found, the entire abdominal cavity was well irrigated with hot sublimate solution, 1 to 5000, and afterwards thoroughly drained, and then abdominal wound closed with large silk for retentive sutures, catgut for peritoneum, muscular and superficial cutaneous. No drainage tube was used. Iodoformized gauze, sublimate gauze, absorbent cotton with firm abdominal bandage constituted the dressing.

Catheterized six hours after operation, some bloody urine drawn off; second catheterization urine much clearer, and before time for my regular visit, the patient had passed his urine voluntarily which was clear, after which the catheter was dispensed with altogether.

March 3rd, a. m., 24 hours after the operation. Pulse 100, T. 100., p. m., pulse 95, T. 101.

March 4th, a. m., P. 90, T. 99., p. m., P. 96, T. 100.

March 4th, bowels moved by aid of enema. No blood.

March 5th, a. m,, P. 82, T. 99.

March 6th, a. m., P. 76, T. 101.

March 7th, a. m., P. 76, T. 99½. Bowels moved well by saline cathartic.

March 8th, a. m., P. 82, T. 100., p. m., P. 90, T. 102½.

March 9th, a. m., P. 100, T. 101. Removed dressing. Primary union taken place the entire line of incision, but upon removing the sutures I found escaping from the track of one of the deep silk suture a few drops of pus; upon exploring with probe I learned that it led to a pus sack between the muscle layers. I then made a larger opening, irrigated the cavity, introduced a very small drainage tube, which for safety had retained for a week or more, after which it was removed and the opening speedily closed,

Nothing further occurred in the progress of recovery until the 19th, when the temperature rose to 103. Presuming this pyrexia due to some imprudence in diet and a malarial influence, I gave a mild laxative, and several portions of quinine, and in a few days discharged the patient from my care.

At this writing, now eight weeks since the patient's recovery, he is perfectly well, suffering no inconvenience in any way, and performing daily manual labor.

That the patent would have succumbed to the effects of the gun-shot wound without a laparotomy, to me there is not a question or doubt.

The abundance of bloody urine in the peritoneal cavity, the continual leakage of the bladder contents, could but have engendered a septic peritonitis' with all its horrible consequences.

